# *BRCA* Variations Risk Assessment in Breast Cancers Using Different Artificial Intelligence Models

**DOI:** 10.3390/genes12111774

**Published:** 2021-11-09

**Authors:** Niyazi Senturk, Gulten Tuncel, Berkcan Dogan, Lamiya Aliyeva, Mehmet Sait Dundar, Sebnem Ozemri Sag, Gamze Mocan, Sehime Gulsun Temel, Munis Dundar, Mahmut Cerkez Ergoren

**Affiliations:** 1 Department of Biomedical Engineering, Faculty of Engineering, Near East University, Nicosia 99138, Cyprus; niyazi.senturk@neu.edu.tr; 2 DESAM Research Institute, Near East University, Nicosia 99138, Cyprus; gulten.tunceldereboylu@neu.edu.tr; 3 Department of Medical Genetics, Faculty of Medicine, Bursa Uludag University, Bursa 16059, Turkey; berkcandogan@uludag.edu.tr (B.D.); lamiaalieva@uludag.edu.tr (L.A.); ozemri77@yahoo.com (S.O.S.); sehime@uludag.edu.tr (S.G.T.); 4 Department of Translational Medicine, Institute of Health Science, Bursa Uludag University, Bursa 16059, Turkey; 5 Department of Electrical and Computer Engineering, Graduate School of Engineering and Natural Sciences, Abdullah Gul University, Kayseri 38000, Turkey; msaitdundar@erciyes.edu.tr; 6 Medical Imaging Techniques, Halil Bayraktar Vocational Health School, Erciyes University, Kayseri 38039, Turkey; 7 Department of Medical Pathology, Faculty of Medicine, Near East University, Nicosia 99138, Cyprus; gamze.mocan@neu.edu.tr; 8 Department of Histology and Embryology, Faculty of Medicine, Bursa Uludag University, Bursa 16059, Turkey; 9 Department of Medical Genetics, Faculty of Medicine, Erciyes University, Kayseri 38000, Turkey; dundar@erciyes.edu.tr; 10 Department of Medical Genetics, Faculty of Medicine, Near East University, Nicosia 99138, Cyprus

**Keywords:** breast cancer, *BRCA1*, *BRCA2*, variation, artificial intelligence, translational fuzzy logic

## Abstract

Artificial intelligence provides modelling on machines by simulating the human brain using learning and decision-making abilities. Early diagnosis is highly effective in reducing mortality in cancer. This study aimed to combine cancer-associated risk factors including genetic variations and design an artificial intelligence system for risk assessment. Data from a total of 268 breast cancer patients have been analysed for 16 different risk factors including genetic variant classifications. In total, 61 *BRCA1*, 128 *BRCA2* and 11 both *BRCA1* and *BRCA2* genes associated breast cancer patients’ data were used to train the system using Mamdani’s Fuzzy Inference Method and Feed-Forward Neural Network Method as the model softwares on MATLAB. Sixteen different tests were performed on twelve different subjects who had not been introduced to the system before. The rates for neural network were 99.9% for training success, 99.6% for validation success and 99.7% for test success. Despite neural network’s overall success was slightly higher than fuzzy logic accuracy, the results from developed systems were similar (99.9% and 95.5%, respectively). The developed models make predictions from a wider perspective using more risk factors including genetic variation data compared with similar studies in the literature. Overall, this artificial intelligence models present promising results for *BRCA* variations’ risk assessment in breast cancers as well as a unique tool for personalized medicine software.

## 1. Introduction

Early diagnosis is the initial step in medical practice [1]. The integration of artificial intelligence (AI) approaches such as machine learning including fuzzy logic, neural network can transform big data into clinically actionable knowledge [2] and will become the foundation of precision medicine in three ways: quick decision making for clinicians, reasonable source for healthcare systems and individual data for better and precise treatment [3]. In particular, AI has been continuing to improve characterizations in genetic and molecular medicine since it was first conceived by providing knowledge management [4]. This has given rise to evidence-based computerized diagnostic tools, intended to aid physicians in making primary medical decisions and hence early diagnosis, which helps reduce the treatment options and increase survival rate [5]. An artificial intelligence model is used to simplify and accelerate this complex decision-making process. Some of the most important areas in medical research are related to cancer and cardiovascular diseases [4,6,7]. It is based on the complex clinical decision-making method that often accompanies the degree of uncertainty [8].

Breast cancer, as a heterogeneous disease, is the most common cause of cancer-related death in women and affects one in eight women globally [9]. In 2020, 2.3 million women were diagnosed with breast cancer and 685,000 deaths resulted from this disease [10]. Molecular, pathological and clinical characteristics complicate the progression of breast cancer [11]. However, the early detection of breast cancer is an effective method of reducing mortality [12]. Despite its complex aetiology, breast cancer is affected by both environmental and genetic factors. Generally, cancer results from the accumulation of genetic variations known as either somatic or germline. The majority (~70%) of breast cancer cases are sporadic [11]. While 10–30% cases are related to the inherited component, 4–5% cases were related autosomal dominant manner. Familial breast cancers are often seen in families and have been associated with susceptibility genes [13].

*BRCA1* and *BRCA2* are involved in maintaining genome integrity, at least in part, by engaging in DNA repair, cell cycle checkpoint control and even the regulation of key mitotic or cell division steps. Thus, the complete loss of function of either protein leads to a dramatic increase in genomic instability [14]. Women who inherit a deleterious germline *BRCA1* or *BRCA2* mutation face high lifetime risks of developing breast cancer by the age of 80, which are estimated to be 72% and 69%, respectively [15,16]. These women have a higher risk of having a second ipsilateral [17] or contralateral [18] breast cancer after being diagnosed with invasive breast cancer. Women with an inherited mutation in these genes also have a higher risk of developing ovarian cancer [19]. For *BRCA1* mutation carriers, the risk increases significantly between the ages of 30–50, while the risks for *BRCA2* mutations are highest between the ages of 40–60 [15]. *BRCA1*-associated breast cancers have aggressive pathological traits and are mainly hormone receptor-negative, whereas *BRCA2*-associated breast cancers have sporadic characteristics and are predominantly hormone receptor-positive [16,20]. *BRCA1* and *BRCA2* genes alteration are also associate with other cancer types such as ovarian cancer (16.5–27%), prostate cancer (15%), pancreas cancer (2–7%) and possible melanoma [21,22]. The risk of ovarian cancer increases significantly by the age of 36–39 with *BRCA1* mutation carriers and by the age of 44–46 with *BRCA2* mutation carriers. On the other hand, the age range is around 63 for sporadic ovarian cancer [23].

The American College of Medical Genetics and Genomics (ACMG) has recommended a five different variant classification: pathogenic, likely-pathogenic, variant with unknown significance (VUS), likely-benign and benign [24]. The pathogenic variants contribute to the development of diseases [25]. However, a single pathogenic variant may not be sufficient to cause a disease. Likely pathogenic variants have a high likelihood (greater than 90% certainty) of causing disease; however, further evidence will be needed to confirm this assertion of pathogenicity [26]. VUS variants are crucial as the potential effect of the variant in the protein structure is either unknow or rare in the population or has not been registered before [24,25,26]. Thus, identification of VUS variants is important for precise treatment and targeted therapies. This developed artificial intelligence models have been successful in characterization the pathogenicity of VUS variants.

In the literature, many studies have used artificial intelligence models and created risk assessment or early prediction software [27,28,29,30,31,32,33,34,35,36,37]. To the best of our knowledge, this is the first study to assess breast cancer risk using *BRCA1* and *BRCA2* genetic variants using the MATLAB for both fuzzy logic and neural network.

## 2. Materials and Methods

### 2.1. Study Design and Cohorts

A retrospective integrated analysis was performed from two independent breast cancer cohorts from Bursa Uludag University, Department of Genetics and Erciyes University, Department of Medical Genetics, respectively. Sixteen different risk factors were determined for each subject. These risk factors were age, sex, consanguinity, family history, affected number of family number, tumour size, lymph node, degree of malignancy, tumour position, oestrogen receptor hormone, progesterone hormone, *BRCA1* gene variation status, *BRCA2* gene variation status, other gene status, diagnosis and variant classification. Other gene clusters include *BLM*, *BARD1*, *RAD50*, *PALB2*, *MSH2*, *ATM*, *MLH1*, *MRE11A*, *PMS2*, *MUTHY*, *XRCC2*, *ATN*, *CDH1*, *BARD*, *FAM175A*, *EPCAM*, *PKD1*, *STK11*, *NBN*, *MSH2*, *CHEK2*, *MSH6*, *CDH2*, *BRIP1*, *PTEN*, *PIK3CA*, *MEN1*, *TP53* and *RAD51D*. A single pathogenic/likely pathogenic variant within any of these genes is sufficient to associate it as a risk factor. Gene variants have been classified using the Guidelines of the American College of Medical Genetics and Genomics (ACMG) and the Association for Molecular Pathology (AMP) [21]. The study protocol was approved by the ethical review board of Near East University (Application no: YDU/2019/70-840).

### 2.2. Variant Analysis

The raw sequence data (FASTQ) was processed into the variant analysis program (Sophia Genetics, Sophia DDM V5.3.8, Saint Sulpice, Switzerland). Genetic variants within breast cancer-related genes were analysed by community databases NCBI dbSNP (http://www.ncbi.nlm.nih.gov/SNP/, accessed on 13 September 2019), 1000 Genomes Project (http://www.1000genomes.org, accessed on 13 September 2019), Exome Aggregation Consortium (ExAC) (http://exac.broadinstitude.org/, accessed on 13 September 2019) and NHLBI Exome Sequencing Project (ESP) Exome Variant Server (http://evs.gs.washington.edu/EVS/, accessed on 13 September 2019), and those with a frequency of more than 0.5% were eliminated. The effect of the determined variants at the level of protein structure was evaluated with the MutationTaster, Polyphen-2, PolyPhen2 and Sorting Intolerant From Tolerant (SIFT) in silico detection programs. Genomic Evolutionary Rate Profiling (GERP) was used when considering evolutionary conservation across species. Variant analysis and interpretation were performed with ClinVar (https://www.ncbi.nlm.nih.gov/clinvar/, accessed on 13 September 2019, Varsome (https://www.varsome.com/, accessed on 13 September 2019) and HGMD Professional 2020.2 (https://portal.biobase-international.com/cgi-bin/portal/login.cgi?redirecturl=/hgmd/pro/start.php?, accessed on 13 September 2019) databases.

### 2.3. MATLAB and Mamdani’s Fuzzy Inference Method 

MATLAB is a multiple paradigm digital computing software (R2018a) and a fourth-generation programming language. MATLAB is a proprietary programming language developed by MathWorks and is a high-performance language for technical computing [38]. It combines computing, visualization and programming in an easy-to-use environment, where problems and solutions are expressed in familiar mathematical notations [39]. In this study, the fuzzy logic-based artificial intelligence model was developed on this platform using Mamdani’s fuzzy inference method [40].

**Figure 1:**
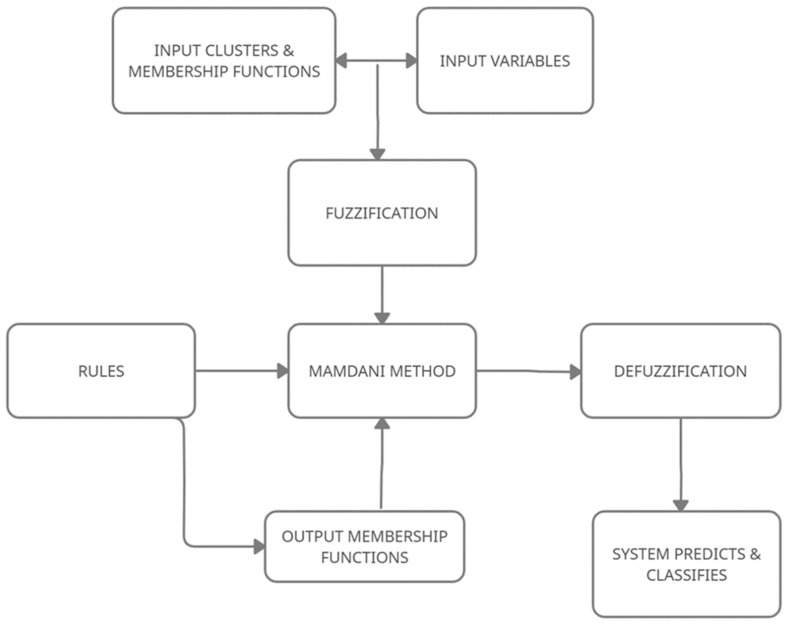
The flowchart of the Fuzzy logic system.

The five well-known main steps were used: (i) The fuzzification of inputs, (ii) Rule values were determined by using fuzzy logic operations, (iii) The implementation of fuzzy cluster logical processors as “and”, “or”, (iv) Collection of results; the combination of fuzzy clusters was represented as output of each rule, (v) Defuzzification, where the system clarified the total fuzzy cluster results and converted them into a single number (Figure 1).

The basis of the fuzzy logic system is the creation of a model that can think and make decisions by using data in input clusters [41]. All rules are evaluated in parallel, and the order of the rules is not important [42]. Our system was developed with 16 input attributes from the dataset of *BRCA* associated breast cancer patients and 1 output attribute with 5 features: pathogenic, likely-pathogenic, VUS, likely-benign and benign. Fuzzification was structured in triangular and trapezoidal membership functions. A Membership Function (MF) is a continuous curve that defines the degree of any numerical variable. The degree of membership is between 0 and 1. Implementation of the Mamdani inference system was made with a rule-based system of 268 rules using if-then statements. The system was characterized by these statements using logical combinations of inputs with an AND operator [43]. The Centroid technique was used for defuzzification and yielded a 95.5% accuracy.

### 2.4. Feed-Forward Neural Network Method

In this study, the neural network-based artificial intelligence model was developed on this platform using feed-forward method [44]. The three well-known main steps were used: (i) The Initialization of network, (ii) Feed-Forward; input values were set and hidden layer values were calculated by using neural network operations, (iii) Backpropagation, where the system clarified the total neural network cluster results and converted them into a single number [45].

An input layer moves in single direction from the input layer to the output layer, with a series of hidden layers and an output layer, each responding to different properties of the data [3]. Therefore, the system learns how to predict the output from the input data.

## 3. Results

### 3.1. Data Collection and Study Design

Sixteen different risk factors were determined for each patient’s data. Each risk factor was divided into sub-groups known as membership functions. Membership functions of each risk factor for both fuzzy logic and neural network models are shown in Table 1.

Data from a total of 932 breast cancer patients were evaluated. 280 patients with genetic variations could be included in the study (Table 2); 268 patients of out 280 were used to train the systems and 12 patients were introduced to systems. These 12 patients were used to test the systems. The remaining 652 patients were therefore not included. It is important to note that that only 22 patients out of 268 were male.

### 3.2. Generating Fuzzy Logic and Neural Network Systems on the MATLAB

As it is crucial to train the complete data to both systems, fuzzy logic and neural network, which includes input, rules and output sections, was generated on MATLAB.

**Figure 2:**
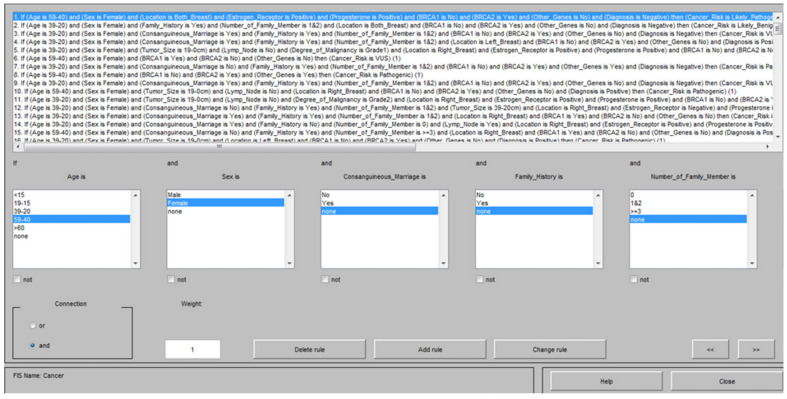
The figure illustrates generated rules section within the Fuzzy Logic system. The upper rectangle box presents an example of the data from 268 patients used that used to train the system. Lower small square boxes show example the parameters (age, sex, consanguineous marriage, family history and number of family members) which were defined as input and membership functions within rule section.

A total of 43 different membership functions from 16 different input clusters were created (Table 1). Each patient’s information was defined as a different rule, which yield as system with different perspectives and possibilities by evaluating 268 different data to give more accurate and sensitive results. For example, the fuzzy logic rules are shown in Figure 2.

**Figure 3:**
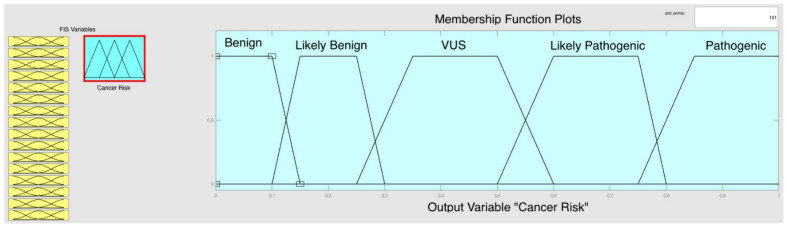
The generated appearance of the output cluster using fuzzy logic interface on the MATLAB. Small-merged yellow boxes illustrate sixteen parameters that were introduced as inputs. The blue box shows the output part and determines five different variant classifications as membership functions. The Y-axis presents membership functions of output which can be determine according to the output score. The X-axis presents values of membership function between 0–1.

Five membership functions, the values of which were given for each membership, were defined at the output section. A classification range was created to determine the variant pathogenicity which was predicted using in silico and variant analysis programs, previously (Table 3). The values in Table 3 were determined by their pathogenic classification according to ACMG [21]. Results were evaluated at the test phase according to given classification values, which were created for the output cluster. Figure 3 shows a fuzzy logic interface on the MATLAB.

**Figure 4:**
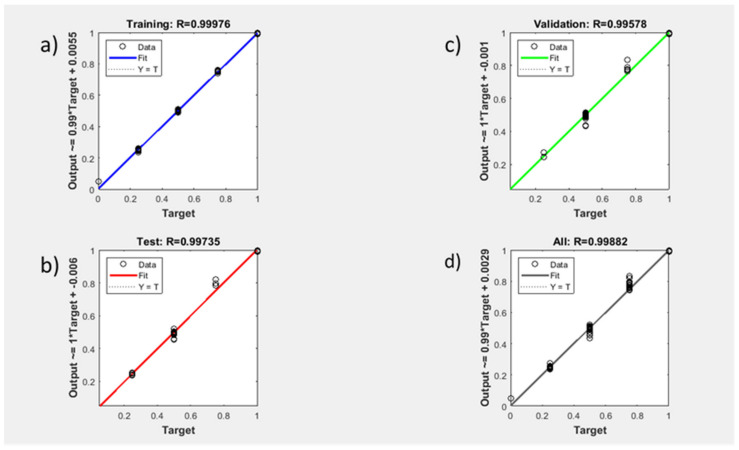
Neural Network regression results of 268 patients. (**a**) The train success of the system using 160 patients (99.9%). (**b**) The test success of the system using 54 patient (99.7%). (**c**) The validation success of the system using remain 54 patients (~99.6%). (**d**) The overall success rate of the system (~99.9%). X-axis represented as output explain regressions data. Y-axis represented as target meaning success ratio between 0–1.

Randomly selected 160 patients were used to train the neural network system, 54 patients were tested for the system and finally, remaining 54 patients therefore used to validate the created data. The training regression (success) was obtained as 99.9% (R = 0.99976). The test success of the system was calculated as 99.7% (R = 0.99735). The validation rate was achieved as ~99.6% (R = 0.99578). Thus, all regression were given as ~99.9% (R = 0.99882) (Figure 4). Thus, this overall result was compatible with the accurate result that obtained by fuzzy logic (95.5%).

### 3.3. Testing the Systems

The designed software systems were tested using an operation test. Six different tests were conducted for 12 different individuals in the test group to check the accuracy and success rates in both systems. These individuals were grouped according to their variant results. It is important to note that these subjects had not previously been introduced to the system. However, the variant classifications were already known. Therefore, the system outcome result confirmed by previously conducted genetic analysis report. Four patients had two different pathogenic variants within either *BRCA1* or *BRCA2.* Two subjects (subject 1 and 2) had the pathogenic *BRCA2* c.7698deIC variant (Table 4) and the other two (subject 3 and 4) had the *BRCA1* C.788dupG (p.Ser264*fs*1) pathogenic frameshift variant (Table 4). After the data for subject 1 and subject 2 were entered, the system calculated values for fuzzy logic were 0.900 and 0.890 and for neural network 0.999 and 0.999, respectively. According to the classification criteria and obtained values, the systems confirmed that both individuals were pathogenic. Subject 3 and subject 4 also had the same pathogenic variant, which gave the same risk scores of for both systems 0.900 and 0.999, respectively. Subject 5 and 6 had the same variant classified as likely pathogenic *BRCA1* c.4070_4071delAA (p.Glu135.7Glyfs*10). While the test was focused on two likely pathogenic variants, we obtained 0.661 for both variants in the fuzzy logic system. However, neural network achieved 0.778 and 0.751, respectively.

On the other hand, the system was tested for variants of unknown significance (VUS) such as *BRCA2* c.9924 A > G (p.Ile3312Val), *BRCA1* c.3368 A > G (p.Lys1290Glu) and *RAD50* c.379 G > A, respectively (Table 4). Therefore, we focused on making the correct estimation of individuals with VUS and possible identification of VUS variants. In the fourth test, we checked the *BRCA2* c.9924 A > G (p.Ile3312Val) VUS variant in both individuals (subject 7 and subject 8). The fuzzy logic system predicted cancer risk scores 0.425 and 0.489 for everyone, respectively. On the other hand, the neural network systems calculated the success rate as 0.502 and 0.449, respectively. Subject 9 and subject 10 both carried the *BRCA1* c.3368 A > G (p.Lys1290Glu) VUS variant according to the ACMG criteria. The value obtained for subject 9 was 0.489. However, subject 10 had a cancer risk score of 0.571 cancer risk, which was within the likely pathogenic threshold in our fuzzy logic system. The neural network system values were 0.505 and 0.503, respectively. Subject 11 and subject 12 carried the same *RAD50* c.379 G > A VUS variant and the fuzzy logic system predicted a value of 0.425 for both individuals, whereas the neural network values were 0.499 and 0.510, respectively.

## 4. Discussion

Artificial intelligence enables cheaper, faster and more practical results in medical diagnosis. As technology develops, the use of artificial intelligence will become more widespread especially in medical diagnosis. Rapid diagnosis and treatment are crucial for the prevention of many diseases, such as cancer in medicine. In this context, artificial intelligence applications have gained importance in recent years. In the last decade, the use of high-throughput sequencing methods accumulated enormous genetic variation data as well as patients’ clinical and laboratory data. For this reason, it is thought that the use of the accumulated data in artificial intelligence applications would determine risk score assessment for the breast cancer which is the most common in women. Therefore, in this study, we aimed to evaluate the risk assessment for *BRCA1-* and *BRCA2-* associated breast cancer using fuzzy logic and neural networks systems.

Machine learning based on artificial intelligence was successfully used to classify cancer risk scores by Kaya and Turk (2020). They used a total of 140 data to test including 130 for test performance analysis and the remaining 10 for status determination [27]. In the current study, 268 different patients’ data were trained in both fuzzy logic and neural network systems. Therefore, broader perspectives were used in both systems for decision-making section whereas the risk of making errors were reduced.

A previous study was focused on cytological and histological image analysis in breast diseases for diagnostic outcomes using the fuzzy logic system on the MATLAB [28].

In 2018, A fuzzy logic system was used to predict breast cancer mortality with only five risk factors such as age, personal history, grade, malignant tumour classification (TNM) stage and multicentricity [29]. Furthermore, Domingo et al., (2019) only used six risk factors on fuzzy logic for predicting the stages of breast cancer [30]. As the variety of risk factors was quite low, they mainly focused on lymph nodes and tumours with a narrow perspective.

Sahria and Mandang (2019) developed a program that could show the risk of breast cancer based on the fuzzy logic method using five histological risk factors for only young women [31]. Controversially, the developed systems in this study can applied any age, gender.

On the other hand, the neural network system was previously proposed to diagnose breast cancer patterns using histological and demographic characteristics, such as Toğaçar et al., (2020) investigated the diagnostic process based on histological image analysis in breast cancer using deep learning and a convolutional neural network giving success rate of 98.80% [32]. In another study, a hybrid deep neural network with artificial intelligence was successfully used to classify breast cancer risk scores by Yan et al., (2020) based on histological image classification and an average accuracy was 91.3% [33]. Zhang et al. (2020) investigated three breast cancer molecular subtypes based on DCE-MRI images using a convolutional neural network [34]. Thus, in this study, 16 different risk factors were used with the aim of obtaining more accurate results affecting breast cancer with a broader perspective to give more significant value than similar studies in the literature.

The most important key point of the study was the risk assessment of two designed different artificial intelligence methods were based on cancer-associate genes and gene variants. Two recent study aimed to predict breast cancer using histopathology and radiology images for *BRCA*-mutation carriers using deep learning [35] and machine learning [36], respectively. This current study mainly focused on gene variant-based risk assessment in cancer.

Genetic variants are classified as pathogenic, likely pathogenic, VUS, likely benign and benign according to ACMG [21]. However, problems arising from the evaluation and diagnosis of VUS variants have been a major challenge for physicians and geneticist today. More importantly, VUS variant carrier cancer patients cannot benefit from treatment processes. A study designed to classify *BRCA* gene related VUS variant in breast cancer using statistical method, previously [37]. In their study, VUS variant classified as either pathogenic or non-pathogenic.

Fuzzy logic and neural network systems in this study were designed and trained to give risk scores to VUS variants using other clinical outcomes of the patient. Therefore, physicians can evaluate VUS variant with given risk score 87 patients with pathogenic, 23 with likely pathogenic, 128 VUS, 29 likely benign and 1 benign *BRCA1* and *BRCA2* gene variants together with 14 other clinical breast cancer risk factors. Moreover, systems were tested for 12 new individuals including two pathogenic (*BRCA2* c.7698deIC and *BRCA1* C.788dupG), one likely pathogenic (*BRCA1* c.4070_4071deIAA) and three VUS (*BRCA2* c.9934 A > G, *BRCA1* c.3368 A > G, *RAD50* c.379 G > A) variants.

In these models, the neural network system overall success rate was achieved as 99.9% whereas training success (99.9%), evaluating validation success (99.6%), test success (99.7%). Therewithal, the fuzzy logic system showed 95.5% accuracy rate. Therefore, as a result, the accuracy rates given by these systems were precisely correct. Software codes will be available Near East University DESAM Research Institute web link (https://desam.neu.edu.tr/, accessed on 17 October 2021).

## 5. Conclusions

Overall, in this study, developed fuzzy logic and neural networks models were found to be successful in predicting correct risk scores for *BRCA1* and *BRCA2* associated breast cancers, especially classifying VUS variants. Thus, we believe that the generated fuzzy logic system will become a good source for the identification of VUS variants in breast cancer diagnosis. To conclude, the artificial intelligence model will provide significant advantages considering an early diagnosis and personalized therapy are vital in cancer.

## Figures and Tables

**Table 1:** Values of membership functions for each input cluster. * VUS: Variant of unknown significance.

Input Clusters (Risk Factors)	Membership Functions	Values [0,1]
Age	<15	0
	15–19	0.25
	20–39	0.5
	40–59	0.75
	≥60	1
Sex	Male	0
	Female	1
Consanguinuty	No	0
	Yes	1
Family History	No	0
	Yes	1
Number of Family Member	0	0
	1 and 2	0.5
	≥3	1
Tumor Size	0–19 cm	0
	20–39 cm	0.5
	≥40cm	1
Lymph Node	Negative	0
	Positive	1
Degree of Malignancy	Grade 1	0
	Grade 2	0.5
	Grade 3	1
Position	Other	0.25
	Right Breast	0.5
	Left Breast	0.75
	Both Breast	1
Estrogen Receptor	Negative	0
	Positive	1
Progesterone	Negative	0
	Positive	1
*BRCA1*	Negative	0
	Positive	1
*BRCA2*	Negative	0
	Positive	1
Other Genes	Negative	0
	Positive	1
Diagnosis	No	0
	Yes	1
Classification	Benign	0
	Likely Benign	0.25
	VUS *	0.5
	Likely Pathogenic	0.75
	Pathogenic	1

**Table 2:** The distribution of the genes among suiTable 268 patients.

Gene	Number
*BRCA1*	61
*BRCA2*	128
*BRCA1* and *BRCA2*	11
Other genes *	68
Total	268

* Other genes: *BLM*, *BARD1*, *RAD50*, *PALB2*, *MSH2*, *ATM*, *MLH1*, *MRE11A*, *PMS2*, *MUTHY*, *XRCC2*, *ATN*, *CDH1*, *BARD*, *FAM175A*, *EPCAM*, *PKD1*, *STK11*, *NBN*, *MSH2*, *CHEK2*, *MSH6*, *CDH2*, *BRIP1*, *PTEN*, *PIK3CA*, *MEN1*, *TP53* and *RAD51D*.

**Table 3:** The table shows the created output cluster for given variant classificiation values. * VUS: Variant of unknown significance.

Membership Functions of Output Cluster	Values of Membership Functions
Benign	0
Likely Benign	0.25
VUS *	0.5
Likely Pathogenic	0.75
Pathogenic	1

**Table 4:** a. Obtained results from testing the system. b. Obtained results from testing the system.

**a**						
**Risk Factors**	**Test Subject 1**	**Test Subject 2**	**Test Subject 3**	**Test Subject 4**	**Test Subject 5**	**Test Subject 6**
	**Variant: BRCA2 c.7698deIC**		**Variant: *BRCA1* C.788dupG**		**Variant: *BRCA1* c.4070_4071deIAA**	
	**Classification: Pathogenic**		**Classification: Pathogenic**		**Classification: Likely Pathogenic**	
Age	43	36	44	42	34	33
Sex	Female	Female	Female	Female	Female	Female
Consanguineous Marriage	Unknown	Unknown	Yes	Unknown	Yes	Unknown
Family History	Unknown	Unknown	Yes	Unknown	Yes	Yes
Number of Affected Family Member	Unknown	Unknown	1	Unknown	1	3
Tumor Size	17.5 cm	0–1 cm	Unknown	6.6 cm	Unknown	Unknown
Lymp Node	No	No	Unknown	No	Unknown	Unknown
Degree of Malignancy	Unknown	Grade 2	Unknown	Grade 3	Unknown	Unknown
Tumor Location	Right Breast	Right Breast	Right Breast	Right Breast	Right Breast	Unknown
Estrogen Receptor Hormone	Unknown	Unknown	Unknown	Positive	Unknown	Unknown
Progesterone Hormone	Positive	Positive	Unknown	Negative	Unknown	Unknown
*BRCA1*	No	No	Yes	Yes	Yes	Yes
*BRCA2*	Yes	Yes	No	No	No	No
Other Genes	No	No	No	No	No	No
Diagnosis	Yes	Yes	Yes	Yes	Unknown	No
Fuzzy Logic Result	90% (0.900)	89% (0.890)	90% (0.900)	90% (0.900)	66.1% (0.661)	66.1% (0.661)
Neural Network Result	99.9% (0.999)	99.9% (0.999)	99.9% (0.999)	99.9% (0.999)	77.8% (0.778)	75.1% (0.751)
**b**						
**Risk Factors**	**Test Subject 7**	**Test Subject 8**	**Test Subject 9**	**Test Subject 10**	**Test Subject 11**	**Test Subject 12**
	***BRCA2* c.9934 A > G**		***BRCA1* c.3368 A > G**		**Variant: Variant: *RAD50* c.379 G > A**	
	**Classification: VUS**		**Classification: VUS**		**Classification: VUS**	
Age	38	42	58	58	32	40
Sex	Female	Female	Female	Female	Female	Female
Consanguineous Marriage	Unknown	No	Unknown	Unknown	Yes	No
Family History	No	No	Yes	No	No	No
Number of Affected Family Member	0	0	Unknown	0	0	0
Tumor Size	3–4 cm	0.5 cm	Unknown	Unknown	Unknown	30 cm
Lymp Node	No	No	Unknown	Yes	Yes	Yes
Degree of Malignancy	Grade 3	Grade 2	Grade 2	Grade 2	Unknown	Grade 2
Tumor Location	Right Breast	Right Breast	Right Breast	Right Breast	Right Breast	Both
Estrogen Receptor Hormone	Positive	Positive	Positive	Positive	Positive	Positive
Progesterone Hormone	Positive	Positive	Positive	Positive	Positive	Positive
*BRCA1*	Yes	No	Yes	Yes	No	Yes
*BRCA2*	Yes	Yes	No	No	No	No
Other Genes	No	No	No	No	Yes	Yes
Diagnosis	Yes	Yes	Yes	Yes	Yes	Yes
Fuzzy Logic Result	42.5% (0.425)	48.9% (0.489)	48.9% (0.489)	57.1% (0.571)	42.5% (0.425)	42.5% (0.425)
Neural Network Result	50.2% (0.502)	49.9% (0.499)	50.5% (0.505)	50.3% (0.503)	49.9% (0.499)	51.0% (0.510)

## Data Availability

The data presented in this study are available on request from the corresponding author.
